# GaAs Nanowire Growth by MBE with Catalyst Forming Eutectic Points with Both Elements

**DOI:** 10.3390/nano15211664

**Published:** 2025-11-01

**Authors:** Nickolay V. Sibirev, Ilya P. Soshnikov, Igor V. Ilkiv, Evgenii V. Ubyivovk, George E. Cirlin, Igor V. Shtrom

**Affiliations:** 1 Faculty of Physics, St. Petersburg State University, Universitetskaya Emb. 13B, St. Petersburg 199034, Russiae.ubyivovk@mail.spbu.ru (E.V.U.); cirlin@beam.ioffe.ru (G.E.C.); 2 Department of Epitaxial Nanotechnologies, Alferov University, St. Petersburg 194021, Russia; ipsosh@beam.ioffe.ru; 3 Ioffe Institute, St. Petersburg 194021, Russia; 4 IAI RAS, St. Petersburg 198095, Russia

**Keywords:** semiconductor nanowires, vapor–liquid–solid growth, catalyst, doping, growth modeling, structural characterization, crystal phase

## Abstract

A3B5 nanowires are usually grown via the vapor-liquid-solid mechanism. Species from the vapor are incorporated into the nanowires using a catalyst droplet. Typically, the droplet is a low-melting-point eutectic alloy of catalyst and group III metal. This growth imposes a set of limitations on the heterostructure formation and doping. Axial A3B5 heterostructure nanowires obtained via an interchange of group III metals suffer from blurring and kinking. Amphoteric dopants such as Si could act as donors and acceptors, leading to electron-to-hole ratio oscillations along the nanowire. To overcome these limits, the growth with a catalyst, which could dissolve both components of the nanowire, is studied. Tin has a eutectic with both components, As and Ga. This makes the growth of GaAs nanowires with a tin catalyst different from that with standard catalysts. Nanowire growth occurs with at least two types of catalysts, Ga-rich and Ga-poor (As-rich). This article aims to study the nanowire growth with an Sn catalyst. For the first time, the growth of GaAs nanowires using a tin catalyst by molecular beam epitaxy is shown. Tin can serve as a catalyst not only for the chemical growth of GaAs nanowires but also as a nucleation site for their growth. Both compositions of the catalyst are observed. The annealing of a thin film of tin on a Si and GaAs substrate has also been studied. At temperatures below 450 °C, small metal droplets form, while tin dissolves into the substrate at higher temperatures.

## 1. Introduction

The III-V semiconductor materials have a direct band gap, which makes them highly efficient at converting photons into electrons. By combining multiple compounds, these materials can be tuned to different band gaps. The Fermi level can be shifted by doping, but typical growth techniques require materials with a matching lattice structure. This has led to widespread use of heterostructures made from III-V materials for a variety of optoelectronic applications. Although growth techniques of these structures require materials with a matching lattice structure.

The unique nanowire (NW) geometry removes some of the limitations of traditional growth methods, allowing the formation of axial heterostructures even with lattice-mismatched materials [1]. This provides an opportunity for band-gap engineering [2]. In addition, the high surface-to-volume ratio improves light extraction and collection compared to planar structures, making NWs particularly suitable for light emitters and photovoltaic devices [3,4,5]. This explains the continued interest of scientists in III-V NWs.

Most of the III-V NWs are created using the vapor–liquid–solid (VLS) technique with metal catalysts [6,7,8,9,10,11,12]. However, the use of metal seed particles to generate NWs through VLS and similar methods presents challenges for the development of heterostructures that are not encountered in thin film systems [6,12,13,14]. The main difficulty lies in creating sharp interfaces, which is attributed to the solubility of the feed material in the catalyst droplet [6,8,15]. Two main types of challenges are considered: NW kinking [16,17] and heterointerface blurring [8,13,15]. III-V NW doping [14,18] also requires additional studies, since the catalyst material strongly affects the incorporation of dopants. Typically, it is emphasized that Si more easily acts as a p-type dopant in III-V materials than an n-type dopant [14].

Axial III-V NW heterostructures can be easily formed by varying the poorly soluble component of the NW. Usually, metal catalysts such as gold are used in VLS growth, which easily dissolve the metal component and the poorly soluble nitrogen group element. In(As, P), Ga(As, P, N) NW heterostructures were demonstrated [19] and are used in various applications [5].

The driving force of AB NW growth with a foreign catalyst C is supersaturation. Therefore, the equilibrium concentration of at least one component should be negligible. Based on this, we can categorize the growth regimes into A-poor, B-poor, and pure-C regimes, depending on the composition of the droplets. So, a sharp heterointerface on element A could be obtained in an A-poor or pure-C regime, while on element B it could be obtained in a B-poor or pure-C regime. The Pure-C regime is typical of growth with a solid catalyst via the so-called vapor-solid–solid mechanism. The drawback of such a regime is a small growth rate.

Usually, the growth of binary NWs was considered with catalysts that easily dissolve only one component, for example, the growth of GaAs NWs with In or Au catalysts. Ga-In forms a simple eutectic, while Au-Ga has several eutectics. Au-As remains solid up to a temperature of 636 °C, and InAs precipitates from the As solution in In rather quickly. This is the reason why the formation of Ga(As, P) and In(As, P) [5,19] is easier than the (In, Ga)As heterostructure [20]. A possible solution to obtain a good heterostructure in the case of (In, Ga, Al)As is the depletion of the catalyst droplet [8], and switching to a pure-C regime growth.

We propose a different idea: to choose a catalyst that could easily dissolve any component of the III-V NW, but not simultaneously. Such a catalyst for GaAs NW is already known. The growth of Sn-catalyzed GaAs NW by metalorganic vapor-phase epitaxy has already been demonstrated in both regimes. Growth with a high arsenic content in the catalyst droplet was shown at Lund University [21]. Growth with a high gallium content was presented at the Ioffe Institute [22].

**Figure 1:**
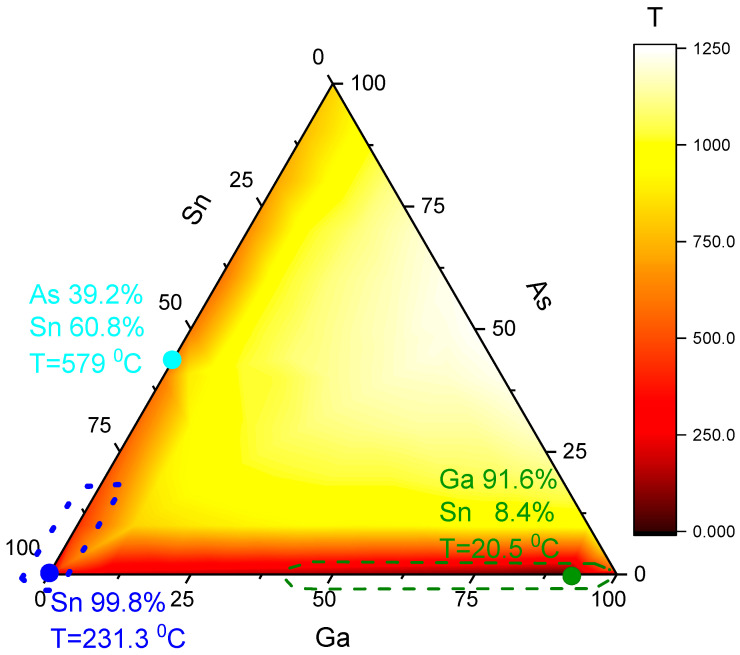
Liquidus surface of the As—Ga—Sn system. Dots pointed to three eutectic points. The dotted line marks the Ga-poor region of Sn-catalyzed NW growth. The dashed line marks the Ga-rich region of Sn-catalyzed NW growth.

The ternary As-Ga-Sn phase diagram is significantly different, see Figure 1, from the regular one for NW growth As-Au-Ga. The As-Ga-Sn diagram has three eutectics: two simple eutectics in the binary diagrams, As-Sn [23] and Ga-Sn [24], and in the vicinity of pure Sn [25,26]. Three eutectic points are marked with circles. The dotted line highlights the hypothetical growth region of NWs with a Ga-poor drop, and the dashed line highlights the growth region with a Ga-rich droplet. The pure-Sn eutectic in the right corner is not very useful for GaAs NW growth. Tin on GaAs substitutes Ga with the formation of Sn_4_As_3_ and liquid Ga-Sn. At higher temperatures, Sn replaces Ga in the GaAs lattice and induces n-type doping of GaAs. The liquidus surface of the ternary diagram is plotted based on the data presented in papers [24,25,26,27].

Therefore, the Sn-GaAs system can be considered as a model for the growth of AB NWs with catalyst C. GaAs NWs can grow from droplets of two different compositions: Ga-Sn and As-Sn. That is, two completely different situations are possible: there is little material A in the catalyst drop or little material B. Therefore, the growth of GaAs with a tin catalyst is also interesting from a basic point of view.

This choice of catalyst also gives the opportunity to obtain sharp and straight heterojunctions in both components of A3B5 NWs. High-quality InAs/GaAs heterointerfaces can be obtained in the As-rich growth regime, when the solubility of the metal component in the Sn-As catalyst droplet is low. Meanwhile, a sharp GaAs/GaP heterointerface is easily reached in the Ga-rich NW growth regime, where P and As group elements are poorly soluble.

It is known that NWs can be grown in the wurtzite (WZ), zinc blende (ZB), and polytype phases [2,5,11,28,29,30,31,32,33,34]. The crystal phase has an important role in the optical and electronic properties of NWs [5,29,30,31]. For example, it is possible to create heterostructures based on NWs, in which the heterogeneous boundary occurs between different phases of the same material [29,30,33]. The droplet surface energy is crucial for determining the crystal phase [34]. The surface energies of liquid gallium [33,34] and tin are quite similar [35], so we expect the same behavior for Sn-catalyzed and self-catalyzed NW [33].

Also, this choice of catalyst gives the opportunity to vary the type of doping without a change in dopant [14]. Si acts as an n-type dopant in the case of the Sn-As catalyst and as a p-type in the case of the Ga-Sn catalyst. The opportunity of n-type doping by tin was also demonstrated [18].

In this paper, the opportunity to grow Sn-catalyzed GaAs NW via molecular beam epitaxy (MBE) is studied.

## 2. Materials and Methods

GaAs NWs were grown on GaAs(111)B or Si(111) with a predeposited thin tin layer in the solid-source MBE system Riber Compact 21 equipped with effusive sources of gallium and arsenic.

First, the substrate was treated with a weak solution of hydrochloric acid to remove the defective oxide layer. Then the 5 nm tin film was deposited by thermal Resistance Evaporator Coater BOC Edwards Auto 500 with oil-free injection and a residual vacuum of at least 5 × 10^−6^ Torr and a substrate temperature of the samples around 80 °C. The deposited film thickness was controlled by optical methods.

Following transfer to the MBE growth chamber, the substrate was heated to a growth temperature ranging from 410 °C to 590 °C. Notably, annealing of the substrate at elevated temperatures prior to growth results in the dissolution of lead into the silicon, thereby inhibiting NW formation. Therefore, NW growth was initiated immediately upon temperature stabilization by the simultaneous opening of the source shutters. Beam equivalent pressure was 3.3 × 10^−7^ Torr for Ga and 1.1 × 10^−5^ Torr for As, which corresponded to a 0.7 ML/s growth rate for 2D GaAs films on. Total growth time was equal to 10 min. In situ reflection high-energy electron diffraction (RHEED) during the growth revealed patterns characteristic of mixed WZ/ZB NWs and the ring-centered pattern of a polycrystalline layer. Growth was terminated by switching off the Ga supply while maintaining the As supply until the substrate temperature dropped to below 300 °C.

The studies of morphological properties of the NW arrays were performed on a field-emission scanning electron microscope (SEM) Supra 25 (C.Zeiss, Oberkochen, Germany) operated at 20 kV, equipped with a microanalysis tool, Ultim (Oxford Instruments Inc., Oxfordshire, UK) for energy dispersive spectrometry (EDS). Investigation of the structural properties and composition of NWs was conducted by methods of transmission electron microscopy (TEM) on a Zeiss Libra 200FE microscope ( Oberkochen, Germany), equipped with an energy-dispersive X-Max X-ray detector. Samples for TEM were obtained by depositing NWs onto carbon film-coated copper grids by gently rubbing the grid against the sample, in most cases breaking the NWs off at the base.

## 3. Results and Discussion

**Figure 2:**
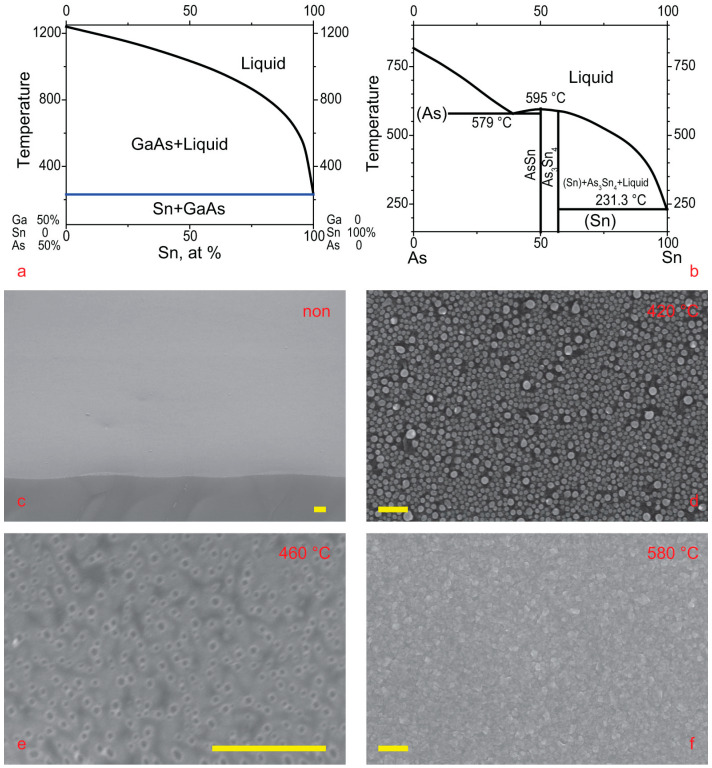
(**a**) Phase diagram for the GaAs-Sn system, (**b**) phase diagram for the GaAs-Sn_3_As_2_ system, (**c**) SEM image of GaAs substrate just after tin layer deposition, (**d**) SEM image of Si substrate after long time etching at 420 °C, (**e**) SEM image of Si substrate after 30 sec etching at 460 °C, (**f**) SEM image of GaAs substrate with tin layer after attempt to NW growth at 580 °C. The scale bar in all images corresponds to 500 nm.

The choice of Sn-catalyzed growth temperature should be based on the triple-phase diagram shown in Figure 1. Ga-rich material can be grown over a wide range of temperatures, from the Sn-Ga eutectic to the GaAs decomposition point. However, Ga-poor growth is only possible within a more limited range of temperatures. The Ga-poor regime of VLS growth can only occur if tin arsenide dissolves in tin. Otherwise, particles of tin arsenide will precipitate from the droplet and disrupt NW growth. The As-Sn eutectic has a relatively high temperature, so the temperature range of As-rich growth is a very narrow temperature range. Pure tin melts at 232 °C, but it does not dissolve well in the Ga-As pair. Even at 600 °C, Sn dissolves a negligible amount of Ga-As; see Figure 2a. The addition of Ga moves the droplet composition from the pure-Sn to the Ga-rich regime. Tin dissolves a reasonable amount of pure As at temperatures greater than 400 °C; see Figure 2b. So interesting that our temperature window is from 400 °C to 600 °C.

Figure 2a,b are cross-sectional views of the ternary diagram shown in Figure 1. In Figure 2a, the diagram intersects along the Sn-GaAs line. In Figure 2b, it intersects along the Sn_3_As_2_-GaAs line.

Initial attempts to grow NWs on Si(111) or GaAs(111) were not successful. NWs did not grow even at 580 °C, as shown in Figure 2f. Therefore, a series of experiments involving the etching of thin tin films on GaAs and Si was conducted.

The tin layer of up to 5 nm was dissolved in the substrate, and the etching pits were clearly visible on the substrate, see Figure 2c,e, even at low temperatures of 450 °C and above. Nevertheless, at temperatures below 425 °C, Sn droplets formed on both Si and GaAs and remained stable for a long time; see Figure 2d. Apparently, this has something to do with the stability of tin arsenides or tin-gallium oxides.

There are two possible ways to initiate high-temperature growth with a tin catalyst: tin oxide deposition and two-stage growth. Both methods have limitations that require further research, which will be performed later.

Tin oxide has a higher melting point than tin, which means it diffuses into the substrate more slowly. For this reason, its diffusion is inhibited before the gallium (Ga) flux is switched on. As a result, the decomposition of tin oxide occurs simultaneously with the formation of Ga-Sn droplets. However, the use of tin oxide requires a study of its decomposition under gallium and arsenic fluxes.

Another opportunity is to change the temperature during the growth. NW growth starts at a low temperature and continues at a high temperature. Two-stage growth requires a study on how to avoid branch formation [36] and NW kinking [16].

The growth of GaAs NWs on a silicon substrate with a tin catalyst is further complicated by the etching of the substrate, which can lead to uncontrolled contamination of the NWs by substrate material. This phenomenon poses a challenge even in the case of lead-catalyzed growth [7] and becomes a significant problem in the case of tin-catalyzed growth. This is because tin is more easily embedded in Si or GaAs compared to lead.

Spontaneous etching of the substrate with tin has led to the fact that attempts to grow NWs at higher temperatures have not been successful. As shown in Figure S1a, NW growth was only observed on torn thin pieces of the substrate. Interdiffusion saturated the silicon piece with tin and stopped. The tin remains in the form of droplets and initiates NW growth. Figure S1b shows such NWs. Despite a high growth temperature of 590 °C, NW growth was initiated on a small shard of the Si substrate, while the rest of the substrate looked similar to Figure 2f, with a rough GaAs layer growing over the silicon substrate.

The growth temperature and substrate were chosen to suppress the dissolution of tin in the wafer and the etching of the substrate by tin. Therefore, the opportunity of growing GaAs NWs with an Sn catalyst is illustrated by growing on a GaAs(111)B substrate at a low temperature of 410 °C. This choice also prevented the incorporation of Si into the NW body. However, reducing the incorporation of the catalyst in the NW and substrate body requires additional research.

**Figure 3:**
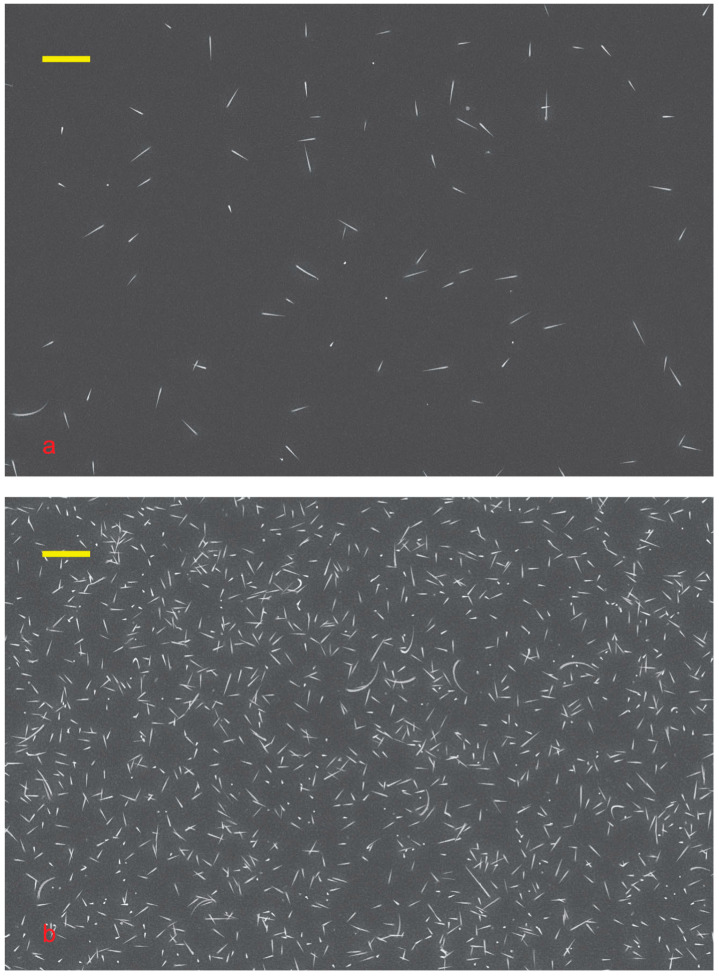
(**a**) SEM image of Sn-catalyzed GaAs NWs, (**b**) SEM image of Pb-catalyzed NWs grown at the same growth conditions. The scale bar in both images corresponds to 2 μm.

It is easy to see that the NWs were grown; see Figure 3. Self-catalyzed growth of GaAs NWs on GaAs is not possible. The arsenic flux is much higher than the gallium flux. Gallium from the droplet diffuses to the substrate and is consumed by the planar growth.

The density of the Sn-catalyzed NW ensemble is low; this is clearly visible from the comparison of Figure 2d and Figure 3a. Even lead-catalyzed NWs ensemble grown with the same amount of catalyst demonstrate greater density, see Figure 3a,b. Despite the growth of Pb-catalyzed NWs was carried out under the same growth conditions, such as temperature, substrate, deposition rate, etc. The growth of lead-catalyzed NW on Si yields a greater NW ensemble density [7]. The gold-catalyzed as well as silver-catalyzed NW growth requires less catalyst [37] and induces a denser NW ensemble. This means that most of the tin dissolves into the substrate.

**Figure 4:**
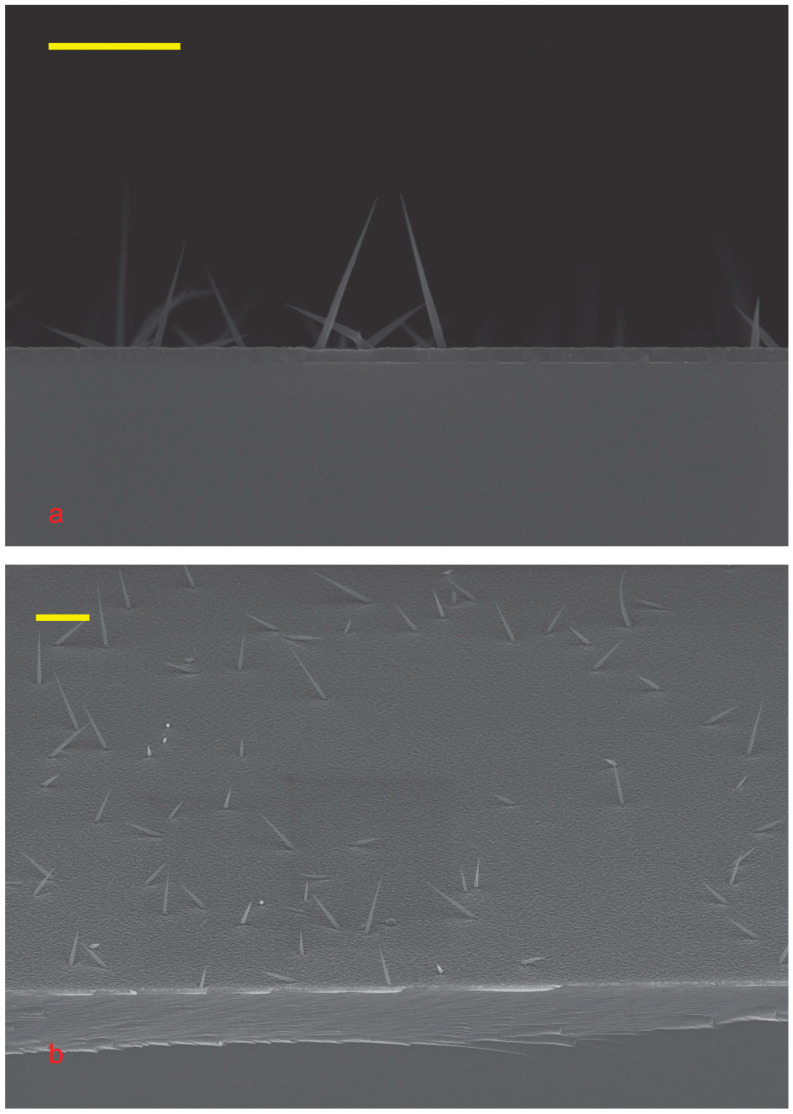
SEM images of Sn-catalyzed GaAs NWs on GaAs at different magnitudes. (**a**) cross section view; (**b**) 54° tilted view. The scale bar in all images corresponds to 1 μm. Growth temperature is 410 °C.

The droplets on the tip are much smaller than the initial ones. Large droplets with a diameter of about 100 nm are clearly visible in Figure 2d, whereas in Figure 3 and Figure 4, all NWs have sharp tips. The decrease in droplets indicates that the tin was incorporated into the body of NW during the growth. Therefore, tin doped our NW.

The NWs are not aligned with the substrate; see Figure 3 and Figure 4. This is due to the presence of tin arsenite (SnAs_2_O_4_) and gallium tin oxide [38] at the base of the NW, which prevents the diffusion of tin into the substrate and alignment. So, the RHEED technique did not allow us to confirm the NW growth in situ.

It is easy to see that the NWs grown have a cone shape; see Figure 3 and Figure 4. The bimodal size distribution of NWs is noteworthy. There are short NWs of about 500 nm, and there are long ones of more than a micron. This may be due to the different compositions of the catalyst droplets. Some droplets have a lot of Ga, and others are Ga-poor. This may also be due to the different sizes of the feeding areas [20,39,40]. Most likely, these mechanisms work together.

Aforementioned above, it was noted that growth was possible with three different droplet compositions: Ga-rich, marked in green; As-rich, marked in cyan; and pure Sn, marked in blue, in Figure 1. The growth temperature is selected below the As-Sn eutectic point. Therefore, growth near the cyan point in Figure 1 is not possible. At temperatures below 500 °C, only two compositions of liquid catalyst are possible. Growth can only occur within the dotted area when using a tin-arsenic catalyst and within the dashed area when using a gallium-tin catalyst.

Such an opportunity is already known for the growth of GaAs NWs with a gold catalyst [12]. The basic description of the NW growth via the VLS mechanism gives the growth of Si NWs with Au catalysts. It claims that growth occurs near the eutectic point. In the case of Au-catalyzed Si NWs, this statement is clearly proven. However, in the case of the growth of GaAs NW with the gold catalyst, the phase diagram is more complicated. The ternary As-Au-Ga phase diagram has a few eutectic points on the Au-Ga side. These eutectic points on the Au-Ga phase diagram correspond to 33.6% Ga, 54.6% Ga, and pure Ga. The first growth experiment was relatively slow and clearly demonstrated that growth proceeds near the Au_2_Ga eutectic point [12,28]. This Ga-poor regime growth of NW requires accounting for the depletion and refill of the liquid phase; nucleation of the next layer is impossible while the previous layer does not cover the entire facet [41].

Further studies demonstrate an opportunity for growth with a Ga rich droplet, where Ga content is greater than 2/3. Even more, in papers [10,11] an opportunity to control the droplet composition was demonstrated. Scientists usually describe this effect as the opportunity to control the NW phase by varying the As/Ga ratio [29]. A higher Au content corresponds to a higher surface energy, which favors the formation of the metastable WZ phase.

The simultaneous existence of two types of catalyst droplets is caused by the initial decay of a thin tin film and the evolution of droplets during NW growth. The initial melting of the thin film leads to a bimodal size distribution of tin droplets, which is typical for decay on non-ideal substrates. Linear defects, such as steps, make the formation of crystallization centers easier, so a lot of small droplets decorate the linear defect, while larger drops form further away. Even on an ideal substrate, coalescence of nanoparticles at the initial stage would broaden the size distribution, as clearly visible in Figure 2d. Large tin drops saturate almost immediately and lead to NW growth with a Ga-poor catalyst, while small tin droplets collect Ga from the substrate surface and initiate NW growth with a Ga-rich catalyst.

The cone diameter of all NWs is about 50 nm, slightly varying for NWs of different heights. This diameter is approximately half the thickness of a 2D gallium arsenide layer. A parasitic bulk layer forms between the NWs, with gallium atoms coming from the flux onto the substrate and incorporating into the buffer layer or diffusing to the NW. Low growth temperature and high arsenic flux make it easier for Ga to incorporate into the substrate. A low NW density reduces the chance of Ga adatoms meeting an NW [20]. Therefore, the NW feeding area is more limited by diffusion than by NW density [39]. In our case, the effective deposition thickness is about 120 nm, with most of the Ga used for the growth of the parasitic 2D layer, whose thickness is about 110 nm.

Most likely, the cone diameter is determined by the radial growth of NWs. The 2D GaAs layer has a constant thickness and no visible pits around the NW. Consequently, the concentration of gallium atoms in the substrate is approximately the same for all NWs. As mentioned previously, the density of NWs is low, as shown in Figure 3, so there is no significant shadow effect. This means that the flow of arsenic atoms towards the base of the NWs is approximately the same for different NWs. The same concentrations of gallium and arsenic lead to similar rates of radial growth for all NWs. The NW growth is initiated by tin particles, so it starts simultaneously for all NWs. The same growth rate and the same growth time result in the same cone diameter of NW. The arsenic flux is close to normal, so the cone diameter is a few times smaller than the 2D layer thickness.

**Figure 5:**
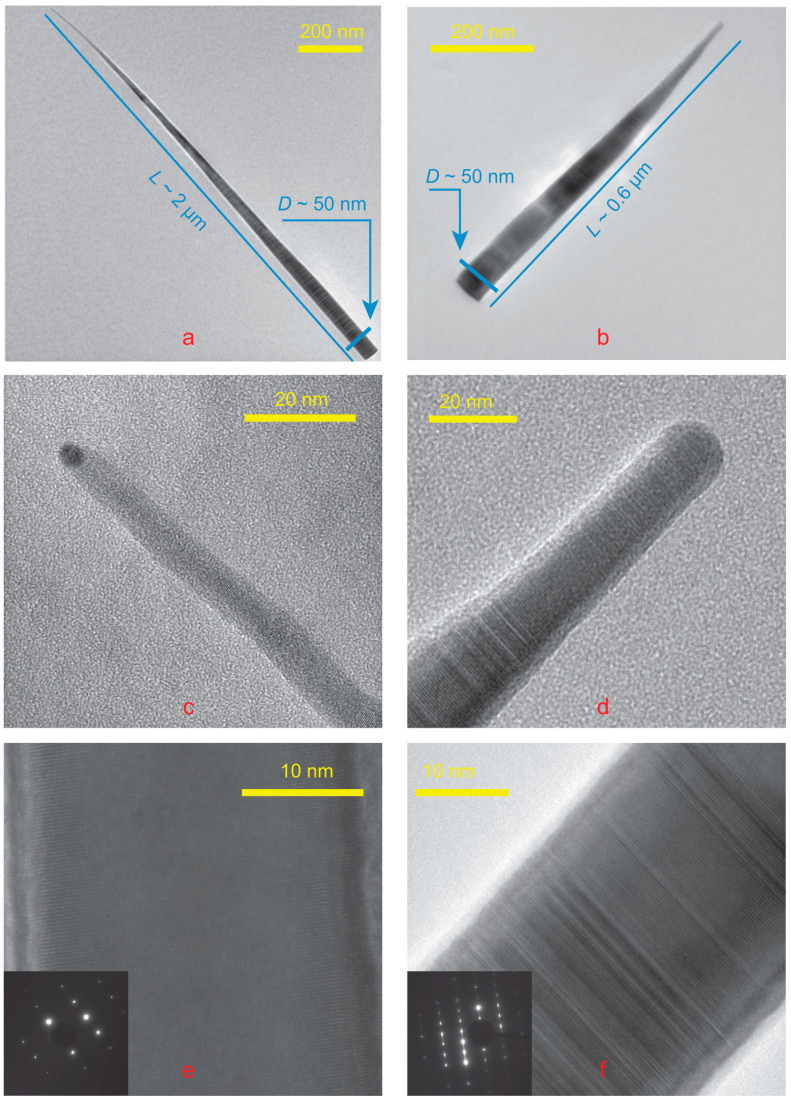
TEM images of two types of Sn-catalyzed GaAs NWs: (**a**,**b**) the scale bars are 200 nm; (**c**,**d**) the scale bars are 20 nm. HRTEM images of Sn-catalyzed GaAs NW tips. (**e**,**f**) The scale bars are 10 nm. HRTEM images of Sn-catalyzed GaAs NWs. (RHEED patterns on the insertions).

HR TEM images were obtained on the field emission gun electron microscope Zeiss Libra 200 FE; see Figure 5. Figure 5a,c corresponds to the long NW, while Figure 5b,d to the short one. Sometimes TEM studies show an elemental contrast at the NW tip compared to the NW body, as seen in Figure 5. The most direct explanation is the presence of a tin droplet on the NW top. The atomic mass of tin is significantly greater than that of gallium and arsenic.

In our case, most of the NWs demonstrate a pure WZ structure, but there were also observed NWs with twins or ZB insertions; see Figure 5. Despite the fact that the crystal structure has a strong effect on the optical properties of NWs [30,31], this is not precisely studied in this paper. The crystal phase of NW is strongly affected by the feeding area or the distance between NW [32,33]. Our experiment design did not allow us to obtain information about the NW surroundings and the crystal phase simultaneously.

The surface energy of tin differs little from the surface energy of gallium [35], so one would expect the same crystal phase as that of self-catalyzed NWs. However, the GaAs phase of NWs can be controlled by growth parameters, as was clearly shown in the paper [33], at nearly the same growth temperature.

The catalyst diameter turned out to be quite small, less than 5 nm. This is significantly less than even the diameter of the NW body; see Figure 5. This does not allow for an accurate element study of the droplet using a TEM setup. The signal from the GaAs NW tip is stronger than from the droplet. An essential part of the beam is focused on GaAs NW tip. Nevertheless, NW growth would obviously not have been possible without this drop, since self-catalyzed growth of GaAs NWs on GaAs(111) is never observed.

The NW growth at our temperatures and catalyst could occur with droplets of two different compositions, Ga-poor and Ga-rich; see Figure 1. The Ga-poor and Ga-rich regions are marked blue and green, respectively. HRTEM allows us to distinguish between these two types of catalysts. The droplet in Figure 5c is Ga-poor, while the droplet in the other Figure 5d is Ga-rich.

The area of the NW sidewalls is more than a hundred times greater than the droplet surface area. The slant height of the NW cone is greater than NW length L, which is about 600 nm for short NW and more than 1100 nm for longer ones; the cone base radius R is about 25 nm. The NW sidewalls area is πRL, more than 40,000 nm^2^. The tip radius of NW r is about 5 nm, so the droplet surface area is about 100 nm^2^. This means that the sidewalls/droplet surface area ratio is much greater than the As/Ga ratio. Therefore, the NW axial growth rate is limited by the As supply. Direct impingement of flux to the droplet is consumed by crystallization and evaporation. In Figure S2, the reader can find bright-field TEM images and NW length statistics based on them.

The solubility of As in liquid Ga is extremely low [25,42]. The amount of As in the droplet is not enough to form a whole monolayer. This leads to the time-scale separation of the ML growth and refill. At the refill stage, As mostly evaporates, while at the ML growth stage, all As is consumed on crystallization. The solubility of arsenic in liquid tin at our growth temperature is about ten percent [23], while the solubility of the Ga-As pair is less than 1% [26], see Figure 2. Therefore, most arsenic is incorporated in NW, while evaporation is negligible. Therefore, NW with Ga-poor droplets grows faster.

The aforementioned radial growth rate is determined by the gallium atoms in the substrate, which is approximately the same for all NWs. That is, an NW grown with an Sn-As catalyst should look more like a prism/cylinder than an NW grown with an Sn-Ga catalyst.

The SEM images show that the aspect ratios of the NWs can be quite different; see Figure 3, which may be explained by this. Sn-As catalyst leads to the formation of longer GaAs NWs than NWs formed with Ga-Sn catalyst. Comparison of TEM images of NWs of both types, see Figure 5, confirms this assumption. In this case, the left part of Figure 5 shows the NW grown with the Sn-As catalyst, and the right part with the Ga-Sn catalyst.

## 4. Conclusions

The first time the growth of GaAs NWs with a tin catalyst via MBE is shown. The presence of droplets on the NW tips is demonstrated. Foreign catalyzed VLS is confirmed. Tin could initiate NW growth not only as a chemical catalyst but also as crystallization centers. The growth with two different compositions of the catalyst is observed. The annealing of thin tin films on silicon and gallium arsenide substrates is studied. Tin could initiate the growth of GaAs NW at temperatures below 450 °C. At high temperatures, Sn rapidly dissolves in the substrate. Growth of GaAs NWs with a tin catalyst requires suppression of the interdiffusion of tin in the substrate.

## Data Availability

Data are contained within the article and Supplementary Materials.

## Supplementary Materials

The following are available online at https://www.mdpi.com/article/10.3390/nano15211664/s1, Figure S1. SEM images of Sn-catalyzed GaAs NWs on a torn thin piece of the Si substrate at different magnitudes. The scale bar in both images corresponds to 1 μm. Growth temperature is 590 °C, (a) The full image of the torn thin piece (b) The enlarged fragment with NWs. Figure S2. The bright field TEM images of NWs (a–c) and NW length statistics according to them (d).
