# Supplementary material for: GaAs Nanowire Growth by MBE with Catalyst Forming Eutectic Points with Both Elements
Source: Nanomaterials (Basel). 2025 Nov 1;15(21):1664. doi: 10.3390/nano15211664 (PMC12608681; doi:10.3390/nano15211664)
Supplement: Supplementary file 1 [file nanomaterials-15-01664-s001.zip › nanomaterials-3953377-supplementary.pdf]

GaAs nanowire growth by MBE with catalyst forming eutectics points with both elements easily dissolving both elements

N.V. Sibirev[1,*], I.P.Soshnikov[3,4], I.V. Ilkiv[1-3] , E.V. Ubyivovk[1,4] , G.E. Cirlin[1-3] , I.V. Shtrom[1,2,*]

[1] St. Petersburg State University, Universitetskaya Emb. 13B, 199034 Saint Petersburg, Russia
[2] IAI RAS, St. Petersburg, 198095, Russia
[3] Alferov University, St. Petersburg, 194021, Russia;
[4] Ioffe Institute, St. Petersburg, 194021,Russia

*Correspondence: n.sibirev@spbu.ru, i.shtorm@spbu.ru

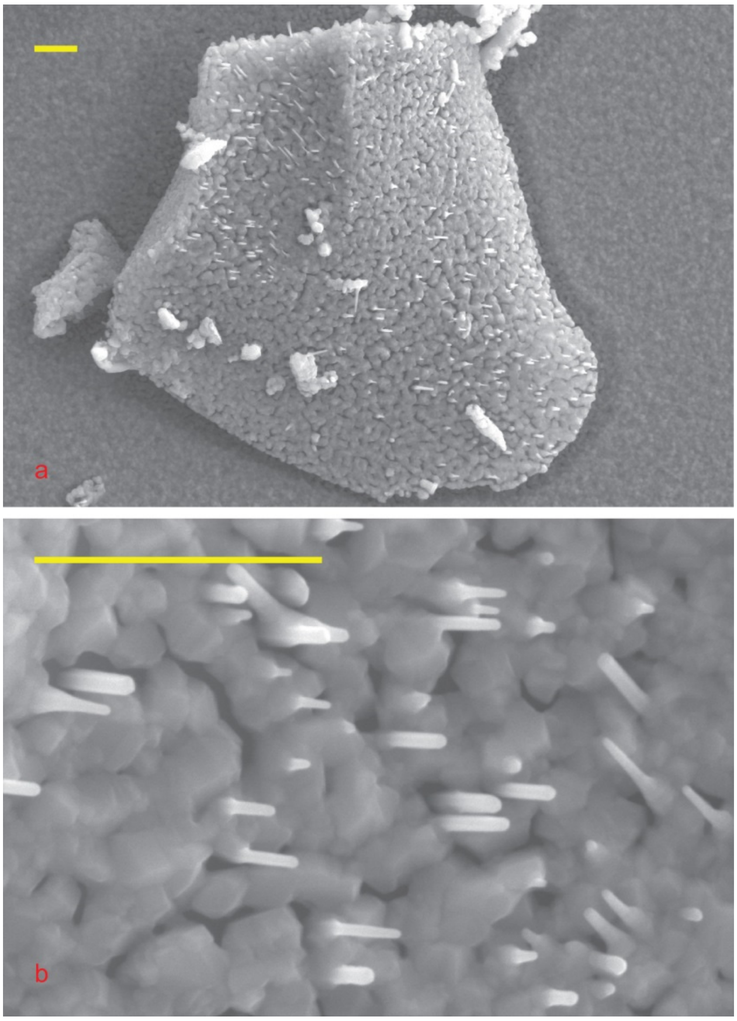

Figure S1. SEM images of Sn-Catalyzed GaAs NWs on torn thin piece of the Si substrate at different magnitude. The scale bar in both images corresponds to 1 μm. Growth temperature is 590 °C. a) The full image of the torn thin piece b) The enlarged fragment with nanowires

As shown in Figure S1a, NWs growth was only observed on torn thin pieces of the substrate. Interdiffusion saturates silicon piece with tin and stopped. The tin remains formed droplets and initiate NW growth. Figure S1b shows such NWs clearly.

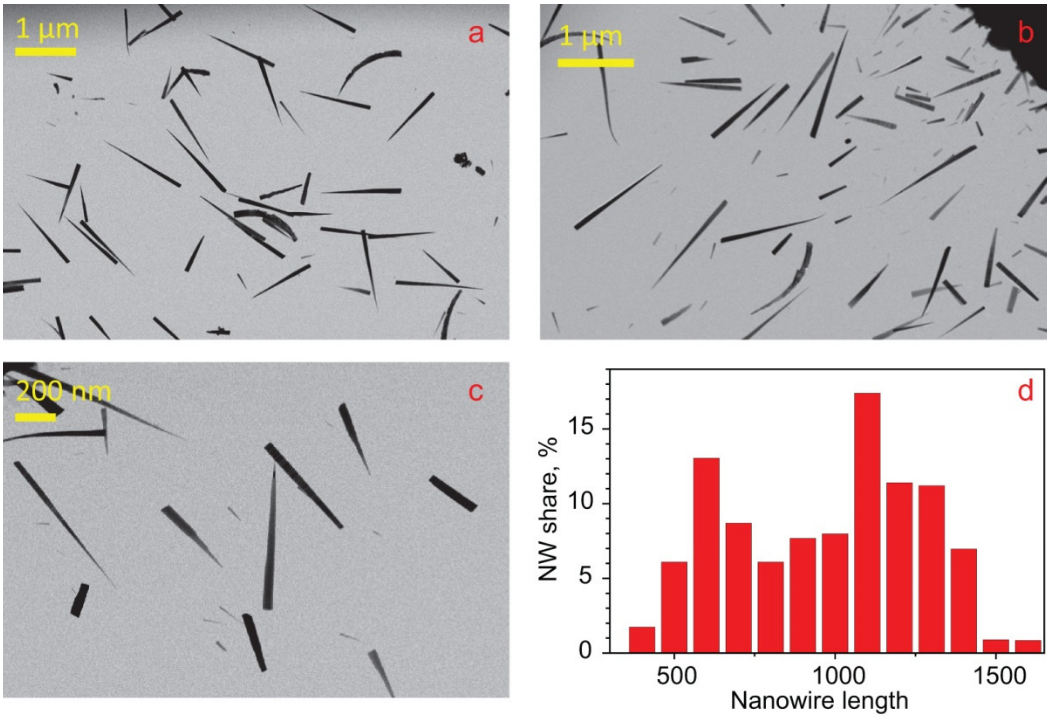

Figure S2. The bright field TEM images of NWs a-c) and NW length statistics according to them d).

Here, the analysis of NWs that were deposited onto a carbon film is presented. The NW length distribution was plotted based on 30 BF-TEM images, about 300 NWs. Broken NWs were excluded based on their cone radius and tip radius, as well as tusk-shaped and kinked NWs. HR-TEM images were taken for eight NWs, which allowed for direct matching of NW length and catalyst droplet composition.

Analysis of SEM images of as-grown NWs is complicated, as our NWs did not epitaxially connect with the substrate. Therefore, NW length measurements require at least two projections. It requires from the microscope operator fixation on particular object, which can unintentionally influence on the length distribution. In our case, the result of length measurements of 20 NWs from SEM images gave a slightly different distribution with two maxima at 500 and 1200 nm.
